# Microneedles and Nanopatches-Based Delivery Devices in Dentistry

**DOI:** 10.15190/d.2020.13

**Published:** 2020-09-30

**Authors:** Panchali Batra, Anika Dawar, Sanjay Miglani

**Affiliations:** ^1^Department of Orthodontics, Faculty of Dentistry, Jamia Millia Islamia, (Central University), New Delhi, India; ^2^Division of Periodontics, Centre for Dental Education and Research, All India Institute of Medical Sciences, New Delhi, India; ^3^Department of Conservative Dentistry and Endodontics, Faculty of Dentistry, Jamia Millia Islamia (Central University), New Delhi, India

**Keywords:** Microfabricated devices, microneedle, nanopatches, oral applications, needle-based devices, oral mucosal patch, dental applications

## Abstract

Needle-based devices are evolving as a promising diagnostic and therapeutic tool in the field of medicine. They can be used for drug delivery, as well as extraction of fluids, for systemic and local effects. The conventional methods of drug delivery require repeated dosing in the oral cavity due to the presence of saliva. Hence delivery systems, such as needle-based devices that could provide sustained release of the drug in the oral cavity, are required. These devices could also be a useful adjunct in diagnosis and therapy of oral cancers, delivering anti-cariogenic and antiplaque agents, for remote monitoring of oral health, and for administering painless and fearless local anesthesia. Since they offer many advantages, such as increased compliance, absence of needle phobia, they are painless, safe, self-applicable and are minimally invasive, they will have a major impact in the field of dentistry. This paper summarizes the various types of needle-based devices and their manufacturing technologies. The manuscript aims to serve as a foundational review that highlights and proposes several current and prospective impactful applications of these devices in various fields of dentistry.

## SUMMARY

*1.* *Introduction*

*2.* *Fabrication*

*3.* *Classification*

*4. Current and prospective applications in dentistry*

- *4.1. Potential applications of needle-based delivery devices in periodontology*

- *4.2.* *Potential applications of needle-based delivery devices in* *oral medicine*

- *4.3. Potential applications of* *needle-based delivery devices* *in oral surgery*

- *4.4. Potential applications of needle-based delivery devices in c* *onservative dentistry, paediatric dentistry and endodontics*

- *4.5.* *Potential applications of needle-based delivery devices in* *orthodontics***

- ** *4.6. Potential applications of* *needle-based delivery devices* *in* *prosthodontics and dental implants***

*5.* *Conclusion*

## 

## 1. Introduction

Needle-based delivery devices are intended to deliver drugs by breaking the barrier of skin or mucosa, thus making the drug readily available to the targeted tissues. Both microneedles and nanopatches have a needle size much shorter than the conventional needles. Microneedle patches consist of sparsely packed miniaturized needles, smaller than 1 mm (hence the name microneedles), mounted on a base which glues to the skin or mucosa. Whereas, nanopatches consist of densely packed needles even smaller than microneedles, of nanoscale dimension. So, the difference between the two is in size and density of needles mounted on the base. Nanopatches were invented to target a greater number of cells and do less damage to the tissues because of their smaller size^1^.

The first concept of microneedles was patented by Gerstel and Place from Alza Research in the 1970s in the US^2^. But because they could not be commercially produced, it took another 25 years to do mass production by economically feasible methods. The nanopatches were invented much later by Professor Mark Kendall from the University of Queensland, Australia^3^.

These devices have the advantage of being less painful and creating less needle phobia in patients, thus increasing patient compliance. Not only this, they could deliver drugs to the specific cells and tissues leading to faster action, more bioavailability, and could also be self-administered. Though rarely, they could elicit an allergic reaction or the miniaturized needles might break. There is a lot of research being done to make these technologies safer and economical so that they could act as agents for drug delivery, fluid extraction, and microdevices for diagnosis and therapeutics. Most of the research in this technology has been done in the medical field, and its potential in dentistry still needs to be explored. This paper attempts to highlight the basics of microneedles and nanopatches, their current applications and suggests some new ideas for possible future applications of this technology in various fields of dentistry.

## 2. Fabrication

Many materials and fabrication techniques have been used for the manufacture of microneedles (Table 1). The choice of the material depends on the design and properties that are desired, such as strength, permeability, and flexibility of the material, which further depends on which tissues one is targeting. The material selected for fabricating solid microneedle should have enough strength and tip sharpness to pierce the targeted tissues.

**Table 1:** Materials and fabrication techniques used for microneedles^4,5^

Type of Microneedles	Materials	Fabrication Technique
Solid	Silicon	Dry-etching, wet etching, isotropic dry etching, anisotropic wet etching
	Metals such as: stainless steel, titanium, tantalum, nickel	Laser ablation, laser cutting, wet etching, metal electroplating
	Polymers such as: methylvinylether and maleic anhydride (PMVE/MA), polycarbonate, polymethylmethacrylate (PMMA), poly-lactic-coglycolic acid (PLGA), polyglycolic acid (PGA), polylactic acid (PLA)	Photolithography, electroplating, molding
	Ceramics	Micromolding and sintering
Coated	Same as solid microneedles	above + coating with viscous solution + drug coating
Dissolving	Sugars, polymers, CMC, polymer chondroitin sulfate, polymer dextrin, PVP, PVA, PLGA, fibroin, dextran	Micromolding, ion etching, photopolymerization process ultrasonic welding, fused deposition modelling with 3D‒printing, micromachining
Hollow	Silicon, glass, polymer, metal	3D‒printing, laser wet chemical etching, deep reactive ion etching, micromachining, lithographic molding, X‒ray photolithography, micro-fabrication

## 3. Classification^3-5^

**Figure 1:**
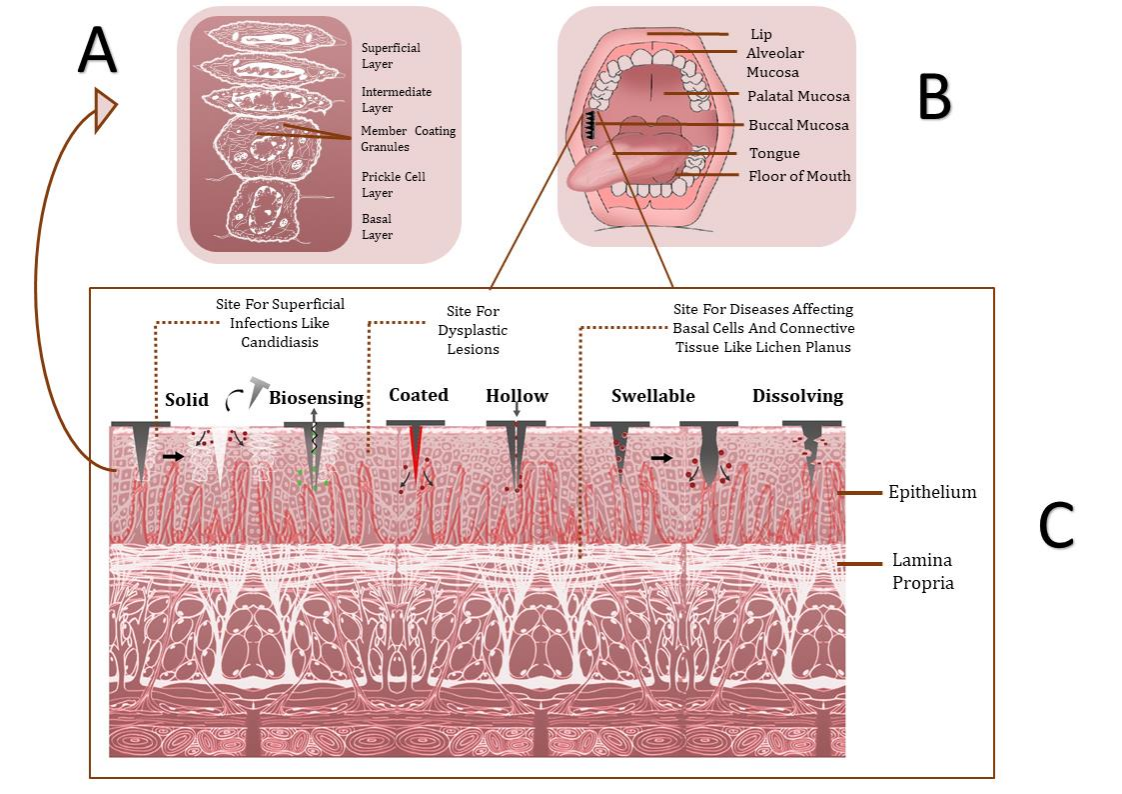
Structure of oral mucosa and the types of microneedles **A. **Structure of oral epithelium; **B.** Needle based patch on buccal mucosa; **C.** Types of microneedles

Microneedles have been classified based on different parameters as shown below. A summary has been provided in Table 2 and Figure 1.

**Table 2:** Classification of microneedles based on structure, needle core, function and design

STRUCTURE	NEEDLE CORE	FUNCTION	DESIGN
a) In plane: the needle is parallel to the base	a) Solid: are used just to create micropores in skin	a) Disposable manner	a) Solid: they are used to create micropores in the skin or create channels for the drug to go inside. The length of the microneedle is decided based on the tissue of target.
b) Out of plane: the needles are perpendicular to the base.	b) Hollow: have a lumen for the transport of materials	b) Multi round responsive	b) Coated: they can be coated with a water-soluble formulation to deliver the drug.
		c) Temperature responsive	c) Dissolving: microneedles can be made of dissolving polymer material with drug encapsulated in the polymer, which dissolves thus releasing the drug at the targeted site.
		d) Glucose responsive	d) Hollow: this microneedle design allows transport of liquid drugs through the bore of the needle and can also be used to withdraw liquids from tissues.
		e) pH responsive	
		f) Swelling - shrinking	
		g) Water soluble	

### 3.1. Structure

a) In-plane: The needle is parallel to the base

b) Out of plane: The needles are perpendicular to the base.

### 3.2. Needle core

a) Solid: is used just to create micropores in the skin

b) Hollow: have a lumen for the transport of materials

### 3.3. Function

a) Disposable manner

b) Multi round responsive

c) Temperature responsive

d) Glucose responsive

e) pH-responsive

f) Swelling-shrinking

g) Water-soluble

### 3.4. Design (Figure 1C)

*Solid:* This microneedle design is used when one needs to create micropores in the skin to create channels for the drug to go inside. The length of the microneedles is decided based on the target cells or target tissue for drug delivery.

*Coated:* The microneedles can be coated with a water-soluble formulation to deliver that drug through the punctured skin and then the microneedle is removed.

*Dissolving:* Microneedles can be made of dissolving polymer material with drug encapsulated in the polymer. When the micropores are formed in the skin the polymer dissolves thus releasing the drug at the targeted site.

*Hollow:* This microneedle design allows transport of liquid drugs through the bore of the needle and can also be used to withdraw liquids from tissues.

## 4. Current and prospective applications in dentistry

The oral environment is constantly bathed by the salivary secretions and any medicine applied on the oral mucosa gets washed off soon and thus requires repeated dosing. Hence delivery systems that could provide sustained release of the drug are required (Figure 1). Needle-based systems offer the advantage that they can be used for drug delivery as well as extraction of fluids for diagnostic and therapeutic purposes and can be used for systemic or local effects. The knowledge of the structure of the oral mucosa (Figure 1A and Figure 1C) is very important to better understand the permeability and hence drug delivery barriers present in the 3 different types of the oral mucosa: masticatory (gingiva and hard palate), specialized (dorsum of the tongue) and lining mucosa (buccal mucosa and floor of the mouth). The permeability of the mucosa depends upon the thickness, keratinization, and lipid composition^6^. Thus, sublingual mucosa is most permeable as it is thin and non-keratinized, followed by buccal mucosa, which is thick and non-keratinized, and lastly, the palatal mucosa which is intermediate in thickness and keratinized. Keratinized gingival and palatal mucosa are good sites for local drug delivery for oral lesions, whereas sublingual mucosa is the best site for systemic absorption of the drugs. The real barrier to drugs is in the upper one third to one-quarter of the epithelium. It is the intercellular material (lipophilic) derived from the membrane coating granules, which slows the passage of hydrophilic drugs (Figure 1A)^6^.

**Figure 2:**
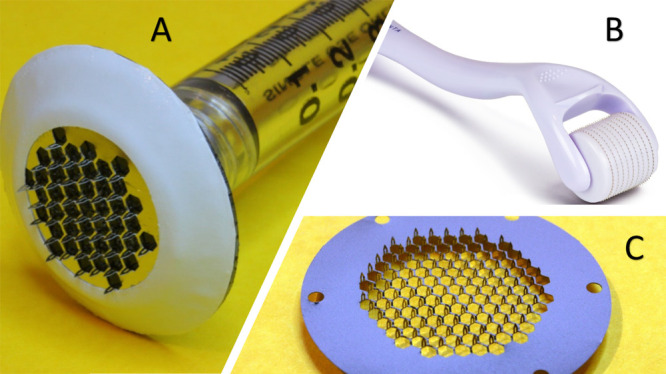
Different needle-based delivery approaches (reproduced with permission) Needle based devices can be fabricated as either: **A.** Pens (AdminPen™ 1200)^7^ **B.** Rollers (e.g. Lolysenta Derma Roller) **C.** Patches (e.g. AdminPatch® 777 microneedle array)^7^

Since the needle-based delivery devices are painless, safe, and can be self-administered, they have a huge potential for application in the field of dentistry. They can be fabricated in any desired shape such as a patch, as a needle, or as a roller depending on the application (Figure 2).

### 4.1. Potential applications of needle-based delivery devices in periodontology

#### a. Enhanced wound healing

Microneedle could be a useful adjunct to periodontal surgery as it could potentially help accelerate the healing process. The minimal superficial bleeding induced by them could set up a wound healing cascade with the release of various growth factors such as platelet-derived growth factor (PGF), transforming growth factor-alpha and beta (TGF-α and TGF-β), connective tissue activating protein, connective tissue growth factor, and fibroblast growth factor (FGF). The needles could also promote neovascularization and neo-collagenesis by migration and proliferation of fibroblasts and laying down of intercellular matrix^8^. Antimicrobial microneedle patches on gingiva could be used to augment periodontal wound healing. Microneedles loaded with antibacterial agents such as green tea and self-sterilizing dissolving nanosilver-loaded patches are being experimented to suppress microbial load at the wound site^9,10^.Also, platelet concentrates such as platelet-rich plasma, platelet-rich fibrin, and growth factors could be administered with the help of microneedles to accelerate healing.

#### b. Improving gingival biotype

Gingival biotype is the thickness of the gingiva in the faciopalatal/ faciolingual dimension. Reduced gingival thickness could lead to attachment loss and marginal tissue recession and thus periodontal disease progression. Gingival biotype also influences the success of restorative and regenerative therapy^11^. The use of microneedles independently or with grafting procedures, based on the principle of accelerated wound healing, could potentially improve the areas with thin gingival biotype. Ozsagir et al. performed a clinical study where they successfully used injectable platelet-rich fibrin (I-PRF) with microneedles to enhance gingival biotype and keratinized gingiva^12^.

#### c. Depigmentation of gingiva

Microneedle roller is already commercially available for skin hyperpigmentation. It breaks the clusters of melanin pigment, stimulates natural wound healing, and production of new collagen and elastin^13^. In the future, a similar principle could be applied for treating gingival hyperpigmentation. Small micro-needle roller designed for oral use could be a promising minimally invasive, simple, painless, and cost-effective treatment modality for gingival depig-mentation as compared to currently used methods such as scalpel surgery, laser ablation, bur abrasion, and electrocautery which often lead to complications such as postoperative pain, bleeding, discomfort, the difficulty of the procedure and delayed wound healing.

#### d. Collecting oral diagnostic fluid

Recently, researchers developed microneedle patches that can easily and painlessly collect interstitial fluid from the skin^14^. Similarly, in dentistry, microneedle patches could be used to collect oral diagnostic fluids such as Gingival Crevicular Fluid (GCF), saliva, and peri-implant fluid. These could be used to identify and quantify various biomarkers, which are important indicators of periodontal disease activity, its progression, and prognostic indicator of therapeutic outcomes. GCF is conventionally collected by paper strips, micropipettes, crevicular washings. However, these methods involve challenges, such as contamination of GCF sample with blood, plaque, and saliva; the amount of GCF collected with these methods is very small; difficulty in retrieving GCF from paper points, etc. Microneedles could potentially help overcome these challenges.

#### e. Administering antiplaque agents

Dental plaque/biofilm is the main etiology for periodontal diseases. Antiplaque agents are particularly important when efficient mechanical plaque control is difficult such as in medically compromised patients, mentally challenged patients, post-surgical cases, orthodontic patients, etc. Novel microneedles patches, which are safe and can be self-administered, loaded with antiplaque agents, could be applied on oral mucosa and gingiva to maintain an optimum level of active agent in the oral cavity for a long time and thus increase the efficacy of these agents.

#### **f.** Administering therapeutics locally

Microneedle patches have emerged as a novel transdermal drug delivery system as they confer advantages such as administration of large molecules, painless admin-istration of the active pharmaceutical ingredient, the first-pass metabolism is avoided, no fear of the needle, ease of administration, targeted drug delivery, enhanced drug efficacy, good tolerability, and rapid and sustained drug delivery^15^. With all these benefits microneedle patches can also be a novel and promising local drug delivery system for periodontal diseases. Microneedles can be used for the administration of antimicrobials, host modulating agents, growth factors, platelet concentrates at specific local sites with periodontal diseases.

### 4.2. Potential applications of needle-based delivery devices in oral medicine

Needle based delivery systems can be used for the following in the field of oral medicine:

#### a. Diagnosis of oral cancers

Oral cancers, such as oral squamous cell carcinoma, are detected late due to their asymptomatic nature. Elevated levels of some biomarkers, such as Cyfra 21-1, tissue polypeptide antigen (TPA), cancer antigen CA-125, MMP-2, MMP-9, and TNF-α, have been associated with oral cancers^16,17^. Needle-based devices could be used for the early detection of these biomarkers.

#### b. Target drug delivery

As mentioned previously, oral mucosa especially the palatal and gingival mucosa are favourable sites for the delivery of drugs for local oral lesions. The site of drug delivery depends on the tissues that are affected. Diseases such as candidiasis affect the most superficial surface of the epithelium, oral dysplasia affects the epithelium itself, lichen planus affects the basal cells, and the adjacent connective tissue (Figure 1C). Thus, the microneedles could be designed depending on the targeted tissue. These microneedles could also be useful in treating premalignant lesions such as leukoplakia.

#### c. Adjunct in cancer chemotherapy

Mucositis and xerostomia are common adverse effects associated with the treatment of cancers. Microneedles could act as agents for the delivery of drugs such as transforming growth factor beta-3 (TGF-b3) or supersaturated calcium phosphate to treat and protect the mucosa.

#### d. Treatment of xerostomia

Xerostomia is commonly encountered secondary to diseases such as Sjogren’s syndrome, diabetes, HIV, or after treatments such as radiotherapy. Drugs such as sodium channel blockers, such as P-552, physostigmine, anhydrous crystalline maltose which can maintain or stimulate the hydration capacity of the oral cavity have shown some potential for topical application^18^. Perhaps a simple microneedle patch could be used to deliver sialagogues in these patients. 

### 4.3. Potential applications of **needle-based delivery devices in oral surgery**

Enhanced wound healing, local drug delivery, administration of local anesthesia are the potential applications of microneedles described in the above sections and are of great use in the field of oral surgery as well. Other possible applications in oral surgery are:

#### a. Analgesic effect in orofacial neuropathic conditions

Neuropathic pain caused by nerve injury is debilitating and difficult to treat. Currently used local anesthetics tend to nonspecifically block both sensory and motor functions. Calcitonin gene-related peptide (CGRP), a neuropeptide released from sensory nerve endings, appears to play a significant role in chronic neuropathic pain^19^. A study in rats has shown an analgesic microneedle (AMN) patch was developed using dissolvable microneedles to transdermally painlessly deliver selective CGRP antagonist peptide for the treatment of localized neuropathic pain^20^. As compared to conventional therapies, the AMN patches produced effective analgesia on neuropathic pain without disturbing the normal motor function of the rat, resulting from the high specificity of the delivered peptide against CGRP receptors^20^. A similar application of AMN patches to oral neuropathic conditions such as trigeminal neuralgia, painful post-traumatic trigeminal neuropathy, burning mouth syndrome, post-herpetic neuralgia, trigeminal neuroma, and other neuropathies related to systemic diseases could provide for an effective, safe, and simple approach to mitigate neuropathic pain with significant advantages over current treatments.

#### b. Management of temporomandibular jaw disorders (TMD)

Patients with TMD presents symptoms such as pain on mouth opening, restricted mouth opening, muscle spasm, clicking, etc. and presents a challenge for oral administration of drugs. Similarly, oral trauma patients with splints, jaw fixation, plating have restricted mouth opening. Microneedle patches can be an effective, safe, and easy way of administering analgesics, anti-inflammatory agents, muscle relaxants in these special cases.

#### c. Alternative to conventional suturing

The technique of suturing post surgically is commonly practiced in the field of oral surgery. Suturing after a surgical incision or dental extraction acts to approximate the edges of the tissue to promote healing. However, suturing has some clinical disadvantages, few to mention are improper approximation can cause tearing of the tissue, the braided suture can accumulate plaque, bacteria and delay wound healing and the suturing process also adds to clinical time spent in surgery. Some alternatives to suturing include the use of adhesive gels, cyanoacrylates, fibrin glue, etc. which overcome the mentioned shortcomings^21,22^. Microneedle patches can provide a sustained and effective method to deliver these agents at a local site. Also, it can act as a delivery agent for hemostatic, adhesives, and sealants after surgical procedure especially in oral sites such as the tongue, the floor of mouth, palate which are difficult to suture. Microneedle patches containing various wound healing promoting agent when applied at surgical sites can augment the healing cascade.

### 4.4. Potential applications of needle-based delivery devices in c**onservative dentistry, paediatric dentistry and endodontics**

#### a. Dental caries

Dental caries is one of the main reasons for adult and pediatric patients to visit a dentist^23^. Despite various advancements, dental caries remains a major health concern and affects nearly 100% of the population in the majority of countries^24^. Dental caries is caused by bacteria that produce acid from the dietary carbohydrates, thereby leading to the demineralization of the hard tissue of the tooth. This effect can be reduced or reversed by the presence of calcium and phosphate in saliva. The amount of these minerals in saliva determines if the tooth shall undergo remineralization or cavitation. Another important mineral that can prevent demineralization is fluoride. Fluoride can form fluoridated hydroxyapatite with enamel hydroxyapatite which can better protect the enamel from acid attacks and demineralization^25^. A higher amount of calcium, phosphate, and fluoride in saliva can reduce the effect of these bacteria and increase the process of remineralization. Microneedles could be used to:

*I. Deliver anti-cariogenic agents:* The current delivery system for the above-mentioned minerals are varnishes, gels, mouthwashes, and toothpaste. These minerals should be continuously present to protect the teeth from acid attack, however, due to the continuous flow of saliva, they get diluted and cleared from the oral cavity thereby reducing their therapeutic effect. Studies have reported that even a small increase in fluoride in the oral cavity can significantly reduce the incidence of caries in pediatric cases^26,27^. Microneedles could be used in pediatric or adult patients with low compliance to oral hygiene procedures for sustained release of fluoride or other remineralizing agents such as Casein phosphopeptide–amorphous calcium phosphate (CPP–ACP) for prevention of caries. 

II. *Streptococcus mutans,* which is present in the dental plaque and is the main causative bacteria for dental caries. Nanoparticles of many metals and metal oxide have shown the potential to destroy the bacteria even in deeper layers of biofilms. Several nanoparticles such as silver nanoparticles, nano zinc oxide, nano gold, nano Chitosan, nanosilver fluoride, etc are been tried for this purpose^28-30^. Microneedle/nanopatches can be an effective way to deliver these nanoparticles and reduce the chances of caries. 

III. *Apart from bacteria and carbohydrates, saliva also plays a major role in the progress of caries.* Salivary factors such as buffering capacity, pH, and salivary flow can also influence the formation and progress of caries^31^. Nanosensors could be a potentially useful tool to monitor these factors in patients with a high caries index.

#### b. Remote monitoring of oral health

Microneedles with sensors could be used to monitor the oral health of soldiers who are posted in far reach areas and have no access to dental treatments.

#### c. Regenerative endodontics

These procedures are designed to replace damaged tooth structures, including dentin and root structures, as well as cells of the pulp–dentin complex. They have gained a lot of interest in the treatment of immature permanent teeth^32^. Several growth factors (PGF, TGFβ, BMP, FGF, VEGF, etc.) play a significant role in cellular functions such as migration, proliferation, and differentiation of dental pulp and stem cells of dentine matrix^33^. Patches with biosensors of growth factors could be used to check the progress while performing regenerative endodontic procedures. 

#### **d.** Administering local anesthesia

Phobia of dental injection affects 1.6% of individuals in the general population^34^. Studies have shown fear of dental needles and the associated pain of its insertion in mucosa harm patient’s psychology, which leads to delay in their dental visits^35^. Microneedles can potentially help in painless administration of lidocaine for periodontal, surgical, and endodontic procedures, thus improving patient compliance. In dentistry, microneedle patches have been used to successfully enhance the effect of topical anesthesia^36^.

### 4.5. Potential applications of needle-based delivery devices in **orthodontics**

#### a. Temporary anchorage devices

TAD’s are often used to augment the anchorage in Orthodontics. Periimplantitis and biofilm formation can lead to failure and loosening of the micro-implants. *Streptococcus spp*, *Lactobacillus casei*, *Candida spp*, and *Porphyromonasgingivalis *are commonly**found microorganisms colonizing on the implant surface^37^. Instead of paper points which are conventionally used to detect colonization, these microneedles can be used as a patch to detect the bacterial growth and release the specific and titrated amount of antimicrobial agent or nanoparticles, which would inhibit that specific bacterial growth.

#### b. Administration of the local anesthetic agents

During the insertion of temporary anchorage devices, local infiltration of anesthetic agents is required which could be done with these patches.

#### c. White spot lesions

White spot lesions and caries are common sequelae during orthodontic treatment. These microneedle patches can be used to deliver fluoride or similar anti-cariogenic substances which will inhibit the development of demineralization during the treatment.

#### d. Anti-sialagogues delivery

Excessive salivary secretions can cause problems in the bonding of brackets to the tooth surface. Anti-sialagogues could be delivered through these patches when the patient is in the waiting area to make bonding more efficient.

#### e. Biomarkers

Biomarkers such as interleukins, tumor necrosis factor-α, transforming growth factor β, proteoglycans, prostaglandin E, and alkaline phosphatases, dentin matrix protein, etc. have been used to either identify the progress of treatment, to assess the response of treatment, or to identify the patients “at-risk”^38^. Biomarkers can be extracted by many means but GCF or saliva is the most non-invasive source. The potential application of needle-based devices to extract and assess biomarkers in the field of orthodontics could be as follows:

I. To assess the response of tissues and to monitor the progress of orthodontic treatment.

II. To assess the levels of salivary alkaline phosphatase which is an indicator of the skeletal maturity of patients in whom growth modification treatment needs to be rendered^39^.

III. To monitor adverse treatment consequences such as root resorption.

IV. To assess the bone activity and turnover in the peri-implant fluid.

V. For assessing the periodontal disease through salivary cytokines^40^.

#### **f.** In patients with cleft lip and palate

Patients with cleft have many issues such as increased caries risk, poor oral hygiene, difficulty in speech, hearing, etc. Needle-based devices could address some of these issues and could be used for remote monitoring of these patients. Maybe if some magnetic particles could be incorporated in these devices, they could be used to align the cleft segments.

#### g. Aligning the jaws

If magnetic patches that could deliver enough force to align the jaws could be made, they could be an alternative to functional appliances. However, many factors such as a mucosal tear, the amount of force generated, the effect on tissues due to prolonged application will have to be evaluated.

### 4.6. Potential applications of **needle-based delivery devices in prosthodontics and dental implants**

#### a. Peri-implantitis

It is an inflammatory pathological reaction around an implant affecting the soft tissue and bony structure, which eventually leads to the loss of the implant. Apart from local debridement and surgical treatment, local application of tetracycline fibers and minocycline microsphere is beneficial in the treatment of peri-implantitis. Microneedle/nanopatches could be an effective way to deliver these antiseptics or antimicrobials to manage the cases of peri-implantitis.

#### b. Denture retention

Denture retention and stability are some of the main determinants in the success of a denture. This can be reduced by several factors such as non-resilient supporting soft tissue, dense fibrous tissue in denture bearing area, xerostomia, highly resorbed residual ridges, etc. Magnets have traditionally been used to increase the retention of overdentures^41^. The same concept can be used with microneedle patches with magnets to increase the retention of loose complete/partial dentures too.

#### c. Gagging

It is commonly encountered in procedures such as recording posterior palatal seal while fabricating complete dentures. Local anesthetics such as lidocaine could be applied via these patches before recording this procedure, to reduce gagging.

#### d. Oral hygiene monitoring and control

Halitosis, poor oral hygiene, development of biofilm overdentures, ulcers, increased caries risk, are common sequelae of wearing a prosthesis. Frequent traveling to dental clinics is many times an issue with geriatric patients. These microneedles could be used to monitors as well as deliver the appropriate medicaments in the susceptible groups.

## 5. Conclusion

Needle-based delivery devices are evolving as a promising diagnostic and therapeutic tool in the field of medicine. Since they offer many advantages, such as increased compliance, absence of needle phobia, painless and safe application, minimally invasive, targeted drug delivery, self-administration, local and systemic applications, they could revolutionize treatment delivery in the field of dentistry. They could be used as an efficient modality for targeted drug delivery in many pathological oral conditions. Also, they have the potential to be used as a diagnostic tool to study oral fluids and for remote monitoring of oral health. However, factors such as the size of the molecule, its residence time, dosage and location, permeability, the structure of the targeted tissues, and the design of the microneedle-based delivery system are key factors in determining their success. Future research and clinical trials can be directed towards designing microneedles, so that they can be used efficiently and safely in the oral cavity for various applications.

## KEY POINTS

**◊ **This review is the first of its kind to explore the application of needle-based devices in the field of dentistry

**◊** Microscale and nanoscale technologies offer promising results in the diagnosis and treatment of diseases and needle-based devices are a relatively new treatment option for oral applications.

**◊** In the field of medicine, these devices have offered many advantages over conventional needles, such as increased compliance, absence of needle phobia, painless and safe application, minimally invasive, targeted drug delivery, self-administration, and local and systemic applications.

**◊** Based on its promising results in medicine, this article proposes some current and prospective applications of these devices in different specialties of dentistry. It also summarizes the various types of needle-based devices and their manufacturing technologies.

**◊** This article opens the doors for future research in designing microneedles, so that they can be used efficiently and safely in the oral cavity applications.

## OPEN QUESTIONS

**◊ **Will microneedles be a clinically relevant and cost-effective alternative to conventional diagnostic and therapeutic modalities in dentistry?

**◊** Do these devices offer the potential for remote monitoring of oral health and implementing preventive measures?

## References

[1] Fernando Germain J P, Chen Xianfeng, Prow Tarl W, Crichton Michael L, Fairmaid Emily J, Roberts Michael S, Frazer Ian H, Brown Lorena E, Kendall Mark A F (2010). Potent immunity to low doses of influenza vaccine by probabilistic guided micro-targeted skin delivery in a mouse model.. PloS one.

[2] Gerstel MS, Place VA (1976). Drug Delivery Device. Google Patents. US Patent No.US3964482A..

[3] Pawar Ravi Gopal (2012). MICRONEEDLES: NOVEL APPROACH TO TRANDERMAL DRUG DELIVERY SYSTEM. Journal of Drug Delivery and Therapeutics.

[4] Yang Jian, Liu Xinli, Fu Yunzhi, Song Yujun (2019). Recent advances of microneedles for biomedical applications: drug delivery and beyond.. Acta pharmaceutica Sinica. B.

[5] Kim Yeu-Chun, Park Jung-Hwan, Prausnitz Mark R (2012). Microneedles for drug and vaccine delivery.. Advanced drug delivery reviews.

[6] Paderni Carlo, Compilato Domenico, Giannola Libero Italo, Campisi Giuseppina (2012). Oral local drug delivery and new perspectives in oral drug formulation.. Oral surgery, oral medicine, oral pathology and oral radiology.

[7] Vadim VY (2010). The AdminPen™ Microneedle Device for Painless & Convenient Drug Delivery. Drug Delivery Technology.

[8] Singh Aashim, Yadav Savita (2016). Microneedling: Advances and widening horizons.. Indian dermatology online journal.

[9] Park So Young, Lee Hyun Uk, Lee Young-Chul, Kim Gun Hwa, Park Edmond Changkyun, Han Seung Hyun, Lee Jeong Gyu, Choi Saehae, Heo Nam Su, Kim Dong Lak, Huh Yun Suk, Lee Jouhahn (2014). Wound healing potential of antibacterial microneedles loaded with green tea extracts.. Materials science & engineering. C, Materials for biological applications.

[10] González García Laura E., MacGregor Melanie N., Visalakshan Rahul Madathiparambil, Ninan Neethu, Cavallaro Alex A., Trinidad Abigail D., Zhao Yunpeng, Hayball A John D., Vasilev Krasimir (2019). Self-sterilizing antibacterial silver-loaded microneedles. Chemical Communications.

[11] Abraham Seba, Deepak K.T., Ambili R., Preeja C., Archana V. (2014). Gingival biotype and its clinical significance – A review. The Saudi Journal for Dental Research.

[12] Ozsagir Zeliha Betul, Saglam Ebru, Sen Yilmaz Berza, Choukroun Joseph, Tunali Mustafa (2020). Injectable platelet‐rich fibrin and microneedling for gingival augmentation in thin periodontal phenotype: A randomized controlled clinical trial. Journal of Clinical Periodontology.

[13] Donnelly Ryan F., Singh Thakur Raghu Raj, Woolfson A. David (2010). Microneedle-based drug delivery systems: Microfabrication, drug delivery, and safety. Drug Delivery.

[14] Wang Ping M., Cornwell Megan, Prausnitz Mark R. (2005). Minimally Invasive Extraction of Dermal Interstitial Fluid for Glucose Monitoring Using Microneedles. Diabetes Technology & Therapeutics.

[15] Bariya Shital H., Gohel Mukesh C., Mehta Tejal A., Sharma Om Prakash (2011). Microneedles: an emerging transdermal drug delivery system. Journal of Pharmacy and Pharmacology.

[16] Nagler R. (2006). Concomitant Analysis of Salivary Tumor Markers--A New Diagnostic Tool for Oral Cancer. Clinical Cancer Research.

[17] Shpitzer T, Hamzany Y, Bahar G, Feinmesser R, Savulescu D, Borovoi I, Gavish M, Nagler R M (2009). Salivary analysis of oral cancer biomarkers.. British journal of cancer.

[18] Sankar V, Hearnden V, Hull K, Juras D Vidovic, Greenberg M S, Kerr A R, Lockhart P B, Patton L L, Porter S, Thornhill M (2011). Local drug delivery for oral mucosal diseases: challenges and opportunities.. Oral diseases.

[19] Benemei Silvia, Nicoletti Paola, Capone Jay G, Geppetti Pierangelo (2009). CGRP receptors in the control of pain and inflammation.. Current opinion in pharmacology.

[20] Xie Xi, Pascual Conrado, Lieu Christopher, Oh Seajin, Wang Ji, Zou Bende, Xie Julian, Li Zhaohui, Xie James, Yeomans David C, Wu Mei X, Xie Xinmin Simon (2017). Analgesic Microneedle Patch for Neuropathic Pain Therapy.. ACS nano.

[21] Ryou Marvin, Thompson Christopher C. (2006). Tissue Adhesives: A Review. Techniques in Gastrointestinal Endoscopy.

[22] Ghosh Abhishek, Nanjappa Madan, Nagaraj Vaibhav, Rajkumar G C (2015). Comparison between stainless steel staples and silk sutures for primary closure of skin in patients undergoing neck dissection: A comparative clinical study.. Contemporary clinical dentistry.

[23] Miglani Sanjay (2020). Burden of Dental Caries in India: Current Scenario and Future Strategies.. International journal of clinical pediatric dentistry.

[24] Petersen Poul Erik, Bourgeois Denis, Ogawa Hiroshi, Estupinan-Day Saskia, Ndiaye Charlotte (2005). The global burden of oral diseases and risks to oral health.. Bulletin of the World Health Organization.

[25] Nguyen Sanko, Hiorth Marianne (2015). Advanced drug delivery systems for local treatment of the oral cavity.. Therapeutic delivery.

[26] Yee R, Holmgren C, Mulder J, Lama D, Walker D, van Palenstein Helderman W (2009). Efficacy of silver diamine fluoride for Arresting Caries Treatment.. Journal of dental research.

[27] Toumba K J, Curzon M E J (2005). A clinical trial of a slow-releasing fluoride device in children.. Caries research.

[28] Keegan Gemma M, Smart John D, Ingram Matthew J, Barnes Lara-Marie, Burnett Gary R, Rees Gareth D (2012). Chitosan microparticles for the controlled delivery of fluoride.. Journal of dentistry.

[29] Batra P, Mushtaq A, Mazumder J, Rizvi MS, Miglani R (2016). Nanoparticles and their Applications in Orthodontics. Adv Dent & Oral Health.

[30] Batra Panchali, Miglani Ragini (2016). Antibacterial properties of retainers with silver nanoparticles.. American journal of orthodontics and dentofacial orthopedics : official publication of the American Association of Orthodontists, its constituent societies, and the American Board of Orthodontics.

[31] Animireddy Dwitha, Reddy Bekkem Venkata Thimma, Vallala Pranitha, Kotha Sunil Babu, Ankireddy Swetha, Mohammad Noorjahan (2014). Evaluation of pH, buffering capacity, viscosity and flow rate levels of saliva in caries-free, minimal caries and nursing caries children: An in vivo study.. Contemporary clinical dentistry.

[32] Murray Peter E, Garcia-Godoy Franklin, Hargreaves Kenneth M (2007). Regenerative endodontics: a review of current status and a call for action.. Journal of endodontics.

[33] Kim Sahng G, Zhou Jian, Solomon Charles, Zheng Ying, Suzuki Takahiro, Chen Mo, Song Songhee, Jiang Nan, Cho Shoko, Mao Jeremy J (2012). Effects of growth factors on dental stem/progenitor cells.. Dental clinics of North America.

[34] Munshi A K, Hegde A, Bashir N (2001). Clinical evaluation of the efficacy of anesthesia and patient preference using the needle-less jet syringe in pediatric dental practice.. The Journal of clinical pediatric dentistry.

[35] Bienvenu O J, Eaton W W (1998). The epidemiology of blood-injection-injury phobia.. Psychological medicine.

[36] Wilson Joseph R, Kehl Lois J, Beiraghi Soraya (2008). Enhanced topical anesthesia of 4% lidocaine with microneedle pretreatment and iontophoresis.. Northwest dentistry.

[37] de Freitas Amanda Osório Ayres, Alviano Celuta Sales, Alviano Daniela Sales, Siqueira José Freitas, Nojima Lincoln Issamu, Nojima Matilde da Cunha Gonçalves (2012). Microbial colonization in orthodontic mini-implants.. Brazilian dental journal.

[38] Kaczor-Urbanowicz Karolina Elżbieta, Deutsch Omer, Zaks Batia, Krief Guy, Chaushu Stella, Palmon Aaron (2017). Identification of salivary protein biomarkers for orthodontically induced inflammatory root resorption.. Proteomics. Clinical applications.

[39] Alhazmi Nora, Trotman Carroll Ann, Finkelman Matthew, Hawley Dillon, Zoukhri Driss, Papathanasiou Evangelos (2019). Salivary alkaline phosphatase activity and chronological age as indicators for skeletal maturity.. The Angle orthodontist.

[40] Jaedicke Katrin M, Preshaw Philip M, Taylor John J (2016). Salivary cytokines as biomarkers of periodontal diseases.. Periodontology 2000.

[41] Moghadam B K, Scandrett F R (1979). Magnetic retention for overdentures.. The Journal of prosthetic dentistry.

